# Potential role of inducible nitric oxide synthase (iNOS) activity in testicular dysfunction following co-administration of alcohol and combination antiretroviral therapy (cART) in diabetic rats: an immunohistochemistry study

**DOI:** 10.1007/s43188-023-00200-5

**Published:** 2023-08-03

**Authors:** Elna Owembabazi, Pilani Nkomozepi, Ejikeme F. Mbajiorgu

**Affiliations:** 1 School of Anatomical Sciences, University of the Witwatersrand, Johannesburg, 2193 South Africa; 2 Department of Human Anatomy and Physiology, University of Johannesburg, Johannesburg, 2028 South Africa; 3 Department of Human Anatomy, Kampala International University, Western Campus, P.O. Box 71, Ishaka-Bushenyi, Uganda

**Keywords:** Diabetes mellitus, Alcohol abuse, Antiretroviral therapy, Inflammation, Oxidative stress

## Abstract

Diabetes, alcohol abuse, and combination antiretroviral therapy (cART) use have been reported to cause multi-organ complications via induction of oxidative stress and inflammation. Moreover, these are the most common factors implicated in male reproductive dysfunctions. This study evaluated testicular oxidative stress, inflammation, apoptosis, and germ cell proliferation in diabetic rats receiving alcohol or cART and their combination. Thirty adult male Sprague Dawley rats were divided into five groups, each consisting of six rats; control, diabetic only (DM), diabetic treated with alcohol (DM + A), diabetic treated with cART (DM + cART), and diabetic treated with both alcohol and cART (DM + A + cART). After 90 days of treatment, the rats were terminated, and the testes were extracted and processed for immunohistochemistry analysis for oxidative stress, inflammatory cytokines, apoptosis, and cell proliferation marker. In comparison to the control, oxidative stress markers, inducible nitric oxide synthase (iNOS), malondialdehyde (MDA), and 8-hydroxydeoxyguanosine (8-OHDG) increased significantly in all treated groups. Expression of testicular proinflammatory cytokines, interleukin-1β, and tumor necrosis factor-α was upregulated in all treated groups, but interleukin-6 was upregulated in DM, DM + cART, and DM + A + cART treated groups and was downregulated in the DM + A treated group. All treated animal groups showed an upregulation of apoptotic marker (caspase 3) and a downregulation of proliferation marker (Ki-67). However, Ki-67 staining intensity significantly increased in treated animals compared to the control. These findings suggest that diabetes, alcohol abuse, cART use, and their combination via iNOS activity upregulation can induce inflammation and oxidative stress in testicular tissue, stimulating germ cell apoptosis and proliferation inhibition leading to failure of spermatogenesis.

## Introduction

Pathogenesis of multi-organ complications associated with diabetes [4], alcohol abuse [16], and cART use [17] is via oxidative stress and inflammation [18, 19]. Oxidative stress and inflammation are interlinked, stimulate the occurrence of one another [14] and incidentally are the commonest factors in male reproductive dysfunctions [20, 21]. Moreover, a high number of reproductive age males are diabetic [1], involved in a lifestyle of alcohol abuse [2], and are on chronic (HIV-infected) combination antiretroviral therapy (cART) or its prophylaxis [3]. Unfortunately, diabetes [1, 4], alcohol [5, 6], and cART [7, 8] have been reported to cause alternations in reproductive hormone levels, testicular structure, and sperm parameters.

Previous studies have demonstrated decreased tubule diameter, reduced height and derangement of germinal epithelium cell layers, spermatogenic cell loss, sloughed epithelium, and karyolysis in diabetic or/and alcohol-exposed or/and cART-treated animals [9, 10]. Corroborating findings reported from seminal fluid analysis, include reduced sperm count, motility, and viability and an increase in abnormal sperm morphology and DNA fragmentation in diabetic condition [1, 4, 11] alcohol consumption [5, 6, 12], and cART treatment [7, 8, 13]. Testicular and spermatozoa alternations have been linked to increased reactive oxygen species (ROS), and oxidative stress [14, 15]. Markedly, nitric oxide synthases (NOS) are established to mediate testicular oxidative stress induction in several testis disorders including cryptorchidism, testicular torsion, varicocele, and toxicity [22, 23].

The three NOS isoforms viz. endothelial NOS (eNOS or NOS3), inducible NOS (iNOS or NOS2), and neuronal NOS (nNOS or NOS1) catalyze the production of nitric oxide from L-arginine [24]. Nitric oxide (NO) is a free radical recognized to play a regulatory role in the process of spermatogenesis at low concentrations [25, 26], but at elevated levels leads to formation of nitrogen-based reactive oxygen species (ROS), which are detrimental to the testicular tissue [24, 27]. Unlike eNOS and nNOS, iNOS is calcium-independent and produces NO in larger quantities than other isoforms. Therefore, upregulation (NO) is clinically important in the induction of testicular oxidative stress [22, 28].

Evidently, the testis is particularly vulnerable to oxidative stress because of high mitochondrial oxygen consumption to support the inherent spermatogenic cell divisions and steroidogenesis [29]. Testicular tissue is further predisposed to oxidative stress because of poor vascularization and relatively high amounts of unsaturated fatty acids [20, 30]. Therefore, based on previous reports which showed that diabetes [31], alcohol [5], and cART [32] can independently induce oxidative stress and inflammation, this study evaluated the testicular effects of co-existence of cART and alcohol abuse in diabetic conditions relative to inducible nitric oxide synthase (iNOS) activity, oxidative stress, inflammation, apoptosis, and cell proliferation.

## Materials and methods

### Chemical and reagents

Streptozotocin (STZ) (S0130) was procured from Sigma-Aldrich Chemical Company (St. Louis, MO, USA) and Atripla, a fixed-dose combination antiretroviral drug (cART) was purchased from Bristol-Myers Squibb and Gilead Sciences (Foster City, CA, USA). The primary antibodies interleukin-1beta (IL-1β) (ab2105), interleukin-6 (IL-6) (ab9324), tumor necrosis factor-alpha (TNF-α) (ab6671), inducible nitric oxide synthase (iNOS) (ab115819), malondialdehyde (MDA) (ab243066), 8-hydroxydeoxyguanosine (8-OHDG) (ab62623), caspase 3 (ab4051), and Ki-67 (ab15580) were purchased from Abcam (Cambridge, MA, USA). The biotinylated goat anti-rabbit (BA-1000) and goat anti-mouse (BA-9200) secondary antibodies, and Avidin–Biotin Complex kit (ABC) (PK-6100) were purchased from Vector Laboratories (Burlingame, CA, USA).

### Ethical clearance

The Animal Research Ethics Committee (AREC) of the University of Witwatersrand (Wits) approved the study protocol with approval number 2018/011/58/C. All experiments were carried out at Wits Animal Research Facility (WARF) per the guidelines of AREC.

### Animal husbandry

In this study, 30 adult male Sprague Dawley rats (10 weeks old; weighing 330–370 g) were used. Every rat was housed individually in a sterile plastic cage at a room temperature of 21–23 °C, with a 12/12-h light/dark cycle, and allowed free access to rat chow and water. Throughout the 90 days treatment duration, the animals freely accessed drinking water or alcohol, and rat chow according to respective treatment groupings.

### Induction of type 2 diabetes

Type 2 diabetes was induced using a modified procedure described by Wilson & Islam, 2012 [33]. Briefly, animals were fed on a 20% fructose reconstituted rat chow diet for two weeks, after which a single injection of freshly prepared 40 mg/kg STZ in 0.05 M (pH 4.5) citrate buffer was administered intraperitoneally. Then, blood glucose (non-fasting) levels were measured 72 h after STZ administration, and rats with glucose levels ≥ 250 mg/dl were regarded diabetic. Once the diabetic state was confirmed in animals, they were placed on a standard rat chow diet.

### Experimental design

The animals were divided into five groups, each with six animals, as follows. Control group, diabetic (DM) group, diabetic animals treated with 10% v/v alcohol daily (DM + A) group, diabetic animals treated with an extrapolated human recommended dose of 23.22 mg/kg of cART [34] in gelatine cubes daily (DM + cART), and diabetic animals treated with both alcohol and cART (DM + A + cART) group. The animals were treated for 90 days, after which the animals were weighed, anesthetized with 240 mg/ml pentobarbitone, and terminated. The testes were then extracted and preserved in 10% neutral buffered formalin for subsequent processing.

### Food and fluid intake

The amount of food and fluid consumed by each rat was recorded daily throughout the experimental period.

### Body weight and gonadosomatic index

The animals were weighed before termination (final body weight) and testis weight was recorded immediately after their extraction. Then, the final body and testis weights were used to calculate the gonadosomatic index, using the formula previously reported by Olasile et al., 2018 [35]

$$Gonadosomatic\;index = \;\frac{{Testis\;weight\;\;\;}}{{Body\;weight}}\; \times 100\;\left( \% \right)$$

### Immunohistochemistry for oxidative stress, inflammatory, apoptosis, and proliferation biomarkers

The harvested and fixed testes were dehydrated sequentially in 70–100% alcohol grades and embedded in molten paraffin wax and sectioned at 5 μm thickness using a Leica RM 2125 rotatory microtome. The sections were floated in a warm water bath (45 °C) for 60 s, then mounted onto silane-coated glass slides for antibody immunolabeling. The sections were dried overnight, followed by deparaffinizing in xylene, hydrating in a series of decreasing alcohol concentrations, and rinsing in running tap water for 5 min. The sections were incubated in citrate buffer overnight in a water bath at 60 °C for antigen retrieval. Thereafter, sections were rinsed in phosphate-buffered saline (PBS) for 5 min, then, immersed in 1% hydrogen peroxide in methanol for 20 min to inhibit endogenous peroxidase. After rinsing in phosphate-buffered saline (PBS) for three changes of five minutes each, 5% normal goat serum was added to the sections to block nonspecific antibody binding. This was tapped off after 30 min, and the primary antibody added subsequently (1:100 for anti-TNF-α and anti-iNOS, 1:200 for anti-IL-1β, anti-IL-6, anti-MDA, and anti-caspase 3, and 1:1000 for anti-8-OHDG and anti-Ki-67) and left overnight (approximately 16 h) at 4 °C. Afterward, the sections were rinsed in PBS and incubated with the secondary antibody (1:1000 biotinylated goat anti-rabbit for the IL-1β, TNF-α, iNOS, caspase 3, and Ki-67 antibodies and 1: 1000 biotinylated goat anti-mouse for IL-6, MDA, and 8-OHDG antibodies) for 30 min. Followed by rinsing in PBS, then avidin–biotin complex (ABC) reagent was added for 30 min. Subsequently, the sections were rinsed in PBS and incubated with 3, 3’-diaminobenzidine tetrachloride (DAB) for five minutes. DAB was then washed off under running tap water for five minutes and the slides were dipped in hematoxylin for one minute to counterstain. Followed by rinsing in running tap water to remove excess stain, dehydration of slides in alcohol series, and coverslip with Dibutylphthalate Polystyrene Xylene (DPX). For the antibodies with immunoreactivity localized to cell nuclei (IL-6, 8-OHDG, caspase 3, and Ki-67), the total number of cell nuclei expressing immunoreactivity were counted in 20 rounded seminiferous tubules for each animal (i.e., 120 tubules for each group) at × 400. The images of antibodies with immunoreactivity localized to both cell nucleus and cytoplasm (IL-1β, TNF-α, iNOS, and MDA) were captured in 144 microscopic fields at × 100 for each group and the ilastik software (v1.3.3; https://www.ilastik.org) was used for image segmentation. Then Fiji software (v1.52e; https://imagej.net/Fiji) was used to quantify immunostaining in the image segments as we previously described [36]. Below is the procedure for quantifying Ki-67 staining intensity.

#### IHC staining intensity quantification

**Fig. 1:**
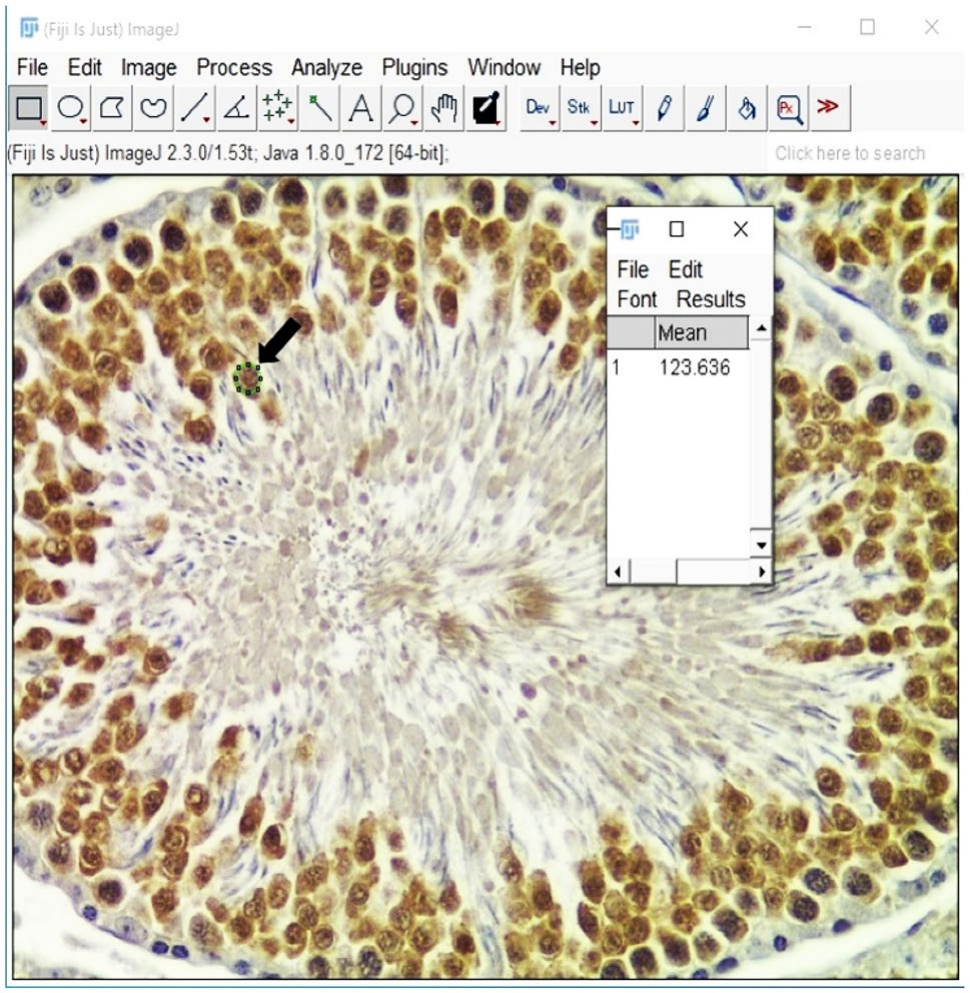
Representative Ki-67 image quantified in Fiji, the mean gray values of a selected region of interest (ROI) illustrated with a thick arrow

The Fiji software (v1.52e; https://imagej.net/Fiji) was used to measure the mean gray values (MGV) of selected stained regions of interest (ROI) as shown in Fig. 1 [37, 38]. Worth noting is that the darker stained areas have a low MGV and the lightly stained areas have a high MGV, thus the staining intensity is equal to the reciprocal of MGV [38]. The steps followed for Ki-67 staining intensity quantification were as follows; opening the image in Fiji: File > Open > Select the image > Open; setting the scale: Analyze > Set scale > Ok; drawing an ROI: Edit > Selection > Specify > Ok; selecting the parameters: Analyze > Set measurement (check the MGV box) > Ok; taking the measurement: Analyze > Measure; opening the next image: File > Open next; choosing the same ROI size and shape: Edit > Selection > Restore selection; proceed to take the measurement: Analyze > Measure; save the results as a CVS file for statistical analysis.

The staining intensity of Ki-67 was calculated as follows. [38]

$$Intensity = \;\frac{1}{MGV}$$

### Data analysis

The data were analyzed using the Windows version of GraphPad Prism 6 and the data was presented as Mean ± SEM. The different group means were compared using one-way analysis of variance (ANOVA), and the Bonferroni post hoc test was performed for multiple comparisons. Deeming *p* < 0.05 value statistically significant.

## Results

### Food and fluid intake

**Fig. 2:**
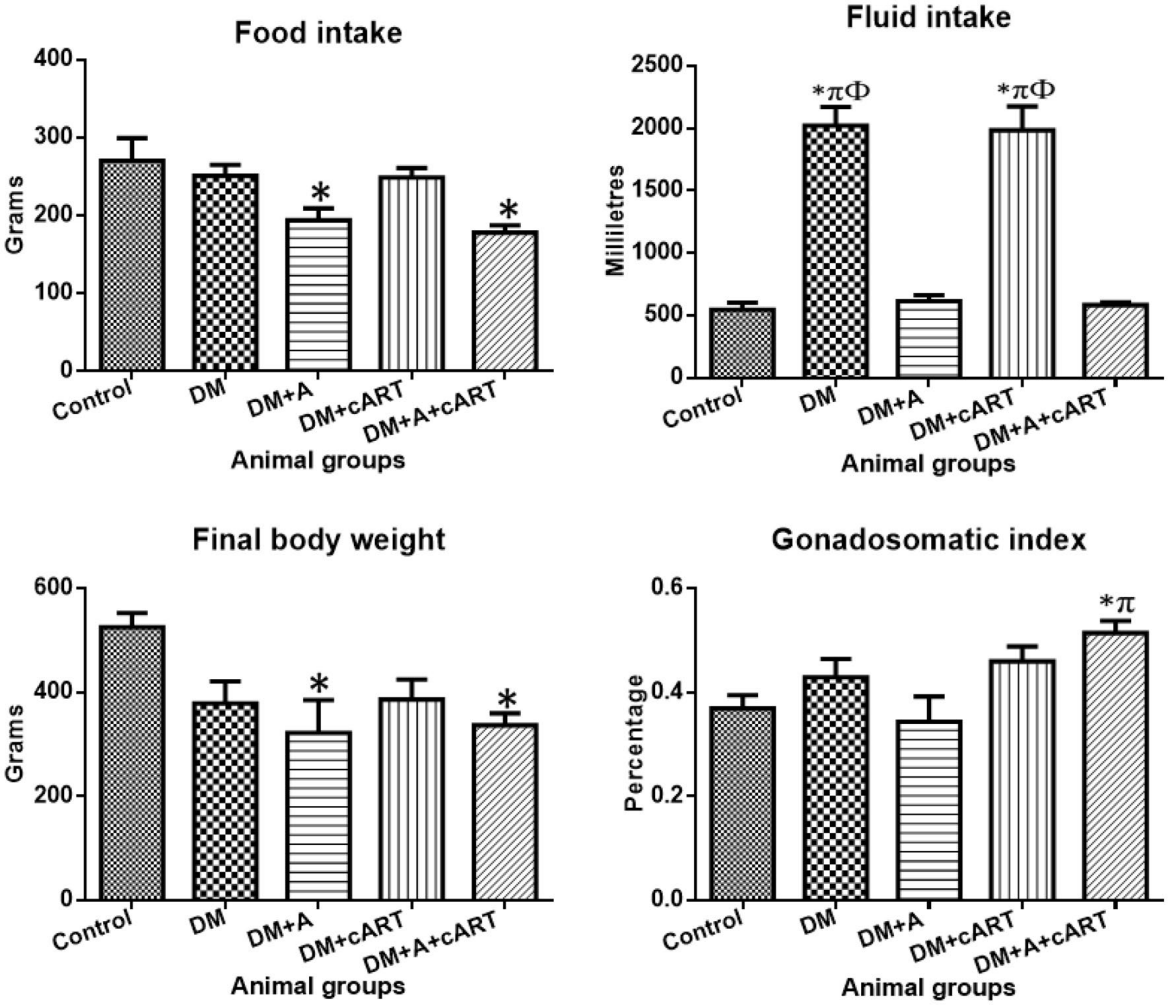
Graphs showing the food and fluid intake, final body weight, and gonadosomatic index. Different symbols *, π, and Ф represent significant differences (*p* < 0.05) as analyzed by a Bonferroni’s multiple comparison test; ‘*’ significantly different compared to control, ‘π’ significantly increased compared to DM + A, and ‘Ф’ significantly increased compared to DM + A + cART. DM, diabetes; DM + A, diabetes and alcohol; DM + cART, diabetes and combination antiretroviral therapy; DM + A + cART, diabetes and alcohol and combination antiretroviral therapy

A general reduction in food intake was recorded in all treated groups relative to the control group but was significant in the DM + A (*p* = 0.0455) and DM + A + cART (*p* = 0.0090) treated groups only (Fig. 2). In comparison with the control, fluid intake increased significantly (*p* < 0.0001) in DM and DM + cART treated groups; however, an insignificant increase (*p* > 0.05) was recorded in DM + A and DM + A + cART treated groups. Further, the fluid intake in DM and DM + cART was significantly increased (*p* < 0.0001) relative to DM + A and DM + A + cART (Fig. 2).

### Body weight and gonadosomatic index (GSI)

All treated groups showed a decrease in final body weight compared to the control, but a significant decrease was recorded in only the DM + A (*p* = 0.0197) and DM + A + cART (*p* = 0.0357) treated groups (Fig. 2). Gonadosomatic index increased significantly in DM + A + cART group relative to control (*p* = 0.0306) and DM + A (*p* = 0.0190). However, the gonadosomatic index of DM + A treated group was insignificantly deceased (*p* > 0.05) compared to the control. Further, a non-significant increase (*p* > 0.05) was recorded in the gonadosomatic index of DM and DM + cART treated groups relative to the control group (Fig. 2).

### Oxidative stress biomarker immunoexpression

**Fig. 3:**
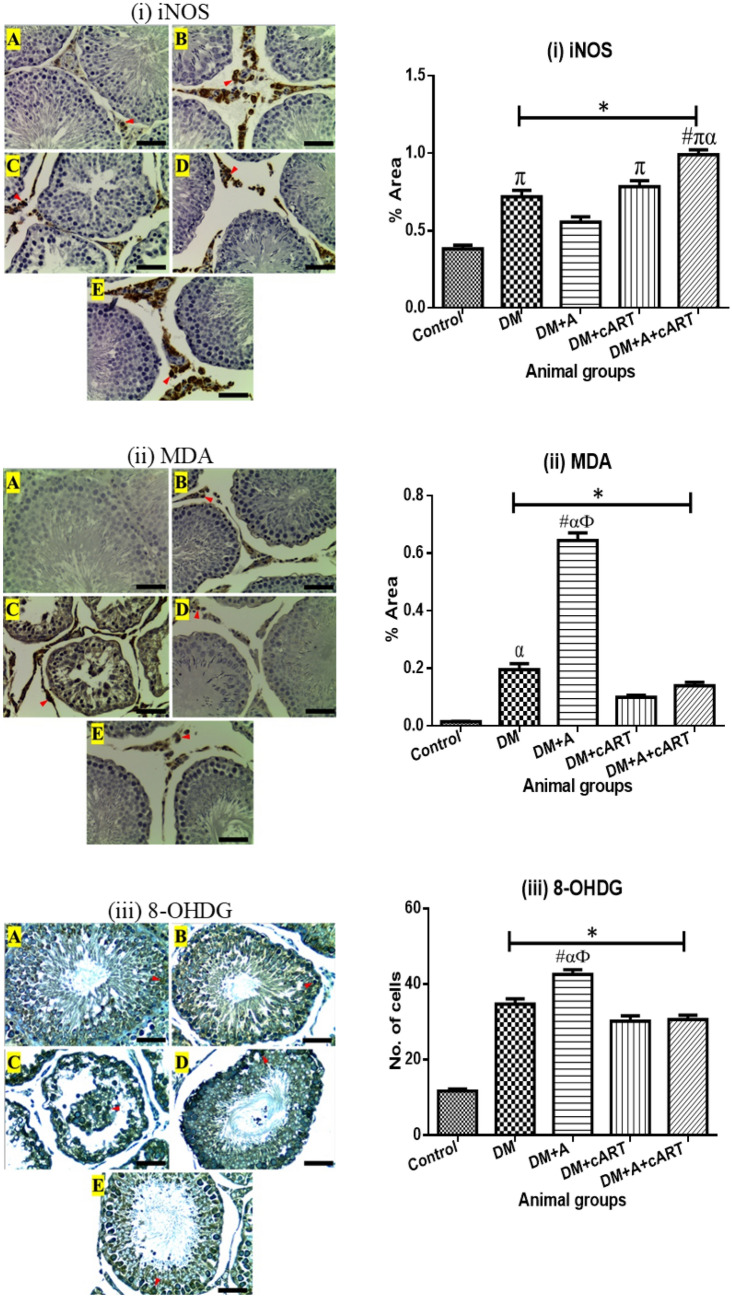
Photomicrographs of oxidative stress markers immunoexpression and respective mean of expression graphs. Representative immunoreactivity is indicated with red arrowheads. (i) iNOS photomicrograph and a graph showing the percentage area of iNOS expression. (ii) MDA photomicrograph and a graph showing the percentage area of MDA expression. (iii) 8-OHDG photomicrograph and a graph showing the number of cells expressing 8-OHDG. Different symbols *, #, π, α, and Ф represent significant differences (*p* < 0.05) as analyzed by a Bonferroni’s multiple comparison test; ‘*’ significantly increased compared to control, ‘#’ significantly increased compared to DM, ‘π’ significantly increased compared to DM + A, ‘α’ significantly increased compared to DM + cART, and ‘Ф’ significantly increased compared to DM + A + cART. Magnification, × 400; scale bar, 50 μm. Key: Images in panel: **a** control group, **b** DM group, **c** DM + A group, **d** DM + cART group, and **e** DM + A + cART group. Treatment groups: DM (diabetes); DM + A (diabetes and alcohol); DM + cART (diabetes and combination antiretroviral therapy); DM + A + cART (diabetes and alcohol and combination antiretroviral therapy)

Expression of iNOS was found in the testicular interstitial cells, Leydig cells, and macrophages of the control and treated groups (Fig. 3). The DM, DM + cART, and DM + A + cART) treated groups had significantly increased iNOS expression compared to the control (*p* < 0.0001 for all) and DM + A (*p* = 0.0005, *p* < 0.0001, and *p* < 0.0001 respectively). The DM + A treated group iNOS expression increased significantly (*p* = 0.0041) relative to the control group. Further, iNOS expression in DM + A + cART group was significantly increased compared to DM (*p* < 0.0001) and DM + cART (*p* = 0.0003) treated groups. Similarly, immunostaining of MDA was detected in the Leydig cells and macrophages (Fig. 3). Compared to the control, the expression of MDA significantly increased in all treated groups (DM: *p* < 0.0001, DM + A: *p* < 0.0001, DM + cART: (*p* = 0.0041, and DM + A + cART: *p* < 0.0001). Also, the MDA expression in DM + A treated group increased significantly (*p* < 0.0001) compared to the other treated groups. Furthermore, MDA expression in DM treated group was significantly increased (*p* = 0.0004) compared to DM + cART. The control and treated groups showed 8-OHDG immunostaining in the spermatogenic cells (Fig. 3). The 8-OHDG immunostaining of all treated groups increased significantly (*p* < 0.0001) compared to control, whilst the expression in the DM + A treated group was respectively significantly increased (*p* < 0.0001) compared to the other treated groups (DM, DM + cART, and DM + A + cART).

### Proinflammatory cytokine immunoexpression

**Fig. 4:**
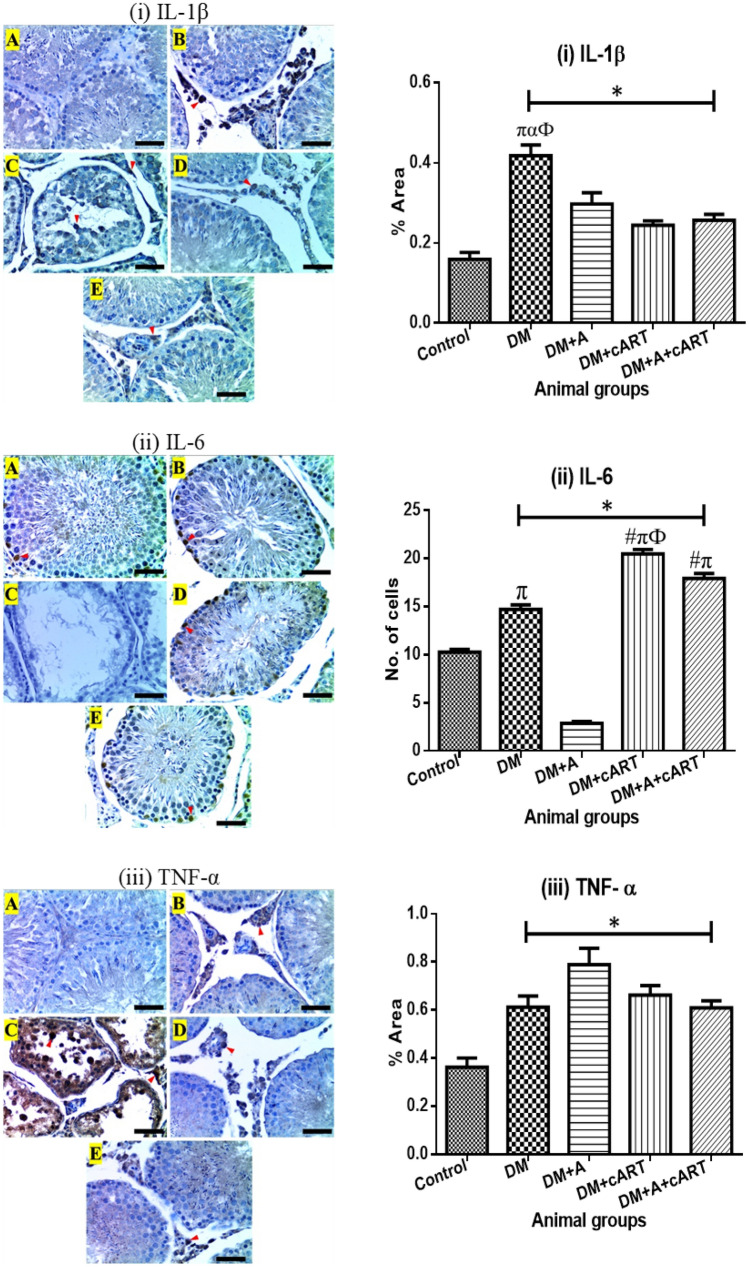
Photomicrographs of cytokine expression and respective mean of expression graphs. Representative immunoreactivity is indicated with red arrowheads. (i) IL-1β photomicrograph and a graph showing the percentage area of IL-1β expression. (ii) TNF-α photomicrograph and a graph showing the percentage area of TNF-α expression. (iii) IL-6 photomicrograph and a graph showing the number of cells expressing IL-6. Different symbols *, #, π, α, and Ф represent significant differences (*p* < 0.05) as analyzed by a Bonferroni’s multiple comparison test; ‘*’ significantly different compared to control, ‘#’ significantly increased compared to DM, ‘π’ significantly increased compared to DM + A, ‘α’ significantly increased compared to DM + cART, and ‘Ф’ significantly increased compared to DM + A + cART. Magnification, × 400; scale bar, 50 μm. Key: Images in panel: **a** control group, **b** DM group, **c** DM + A group, **d** DM + cART group, and **e** DM + A + cART group. Treatment groups: DM (diabetes); DM + A (diabetes and alcohol); DM + cART (diabetes and combination antiretroviral therapy); DM + A + cART (diabetes and alcohol and combination antiretroviral therapy)

The interleukin-1beta (IL-1β) immunostaining was observed in the testicular interstitial cells, macrophages, and Leydig cells of the control and treated groups, except in DM + A treated group that showed IL-1β immunostaining in the germinal epithelium as well (Fig. 4). All treated groups (DM, DM + A, DM + cART, and DM + A + cART) showed significant increases in IL-1β expression in comparison with the control group (*p* < 0.0001, *p* < 0.0001, *p* = 0.0389, and *p* = 0.0095 respectively). Moreso, the expression of IL-1β in the DM group was significantly increased compared to DM + A (*p* = 0.0005), DM + cART (*p* < 0.0001), and DM + A + cART (*p* < 0.0001) treated groups. The interleukin-6 (IL-6) immunostaining in both control and treated groups was detected in Sertoli cells, macrophages, and Leydig cells, except the DM + A group which had immunostaining in only a few Sertoli cells (Fig. 4). For this study, only immunostained Sertoli cells were quantified. The IL-6 expression in DM, DM + cART, and DM + A + cART treated groups increased significantly (*p* < 0.0001) compared to control and DM + A. However, DM + A treated group had a significantly decreased (*p* < 0.0001) IL-6 expression compared to control. Additionally, DM + cART group IL-6 expression was significantly increased compared to DM (*p* < 0.0001) and DM + A + cART (*p* = 0.0001), but the IL-6 expression in DM + A + cART was increased significantly (*p* < 0.0001) compared to DM group. Further, tumor necrosis factor-alpha (TNF-α) immunostaining was similar to that of IL-1β mentioned above. In comparison with control, TNF-α expression increased significantly in all treated groups (DM: *p* = 0.0016, DM + A: *p* < 0.0001, DM + cART: *p* < 0.0001, and DM + A + cART: *p* = 0.0019) (Fig. 4).

### Apoptosis marker, caspase 3 immunoexpression

**Fig. 5:**
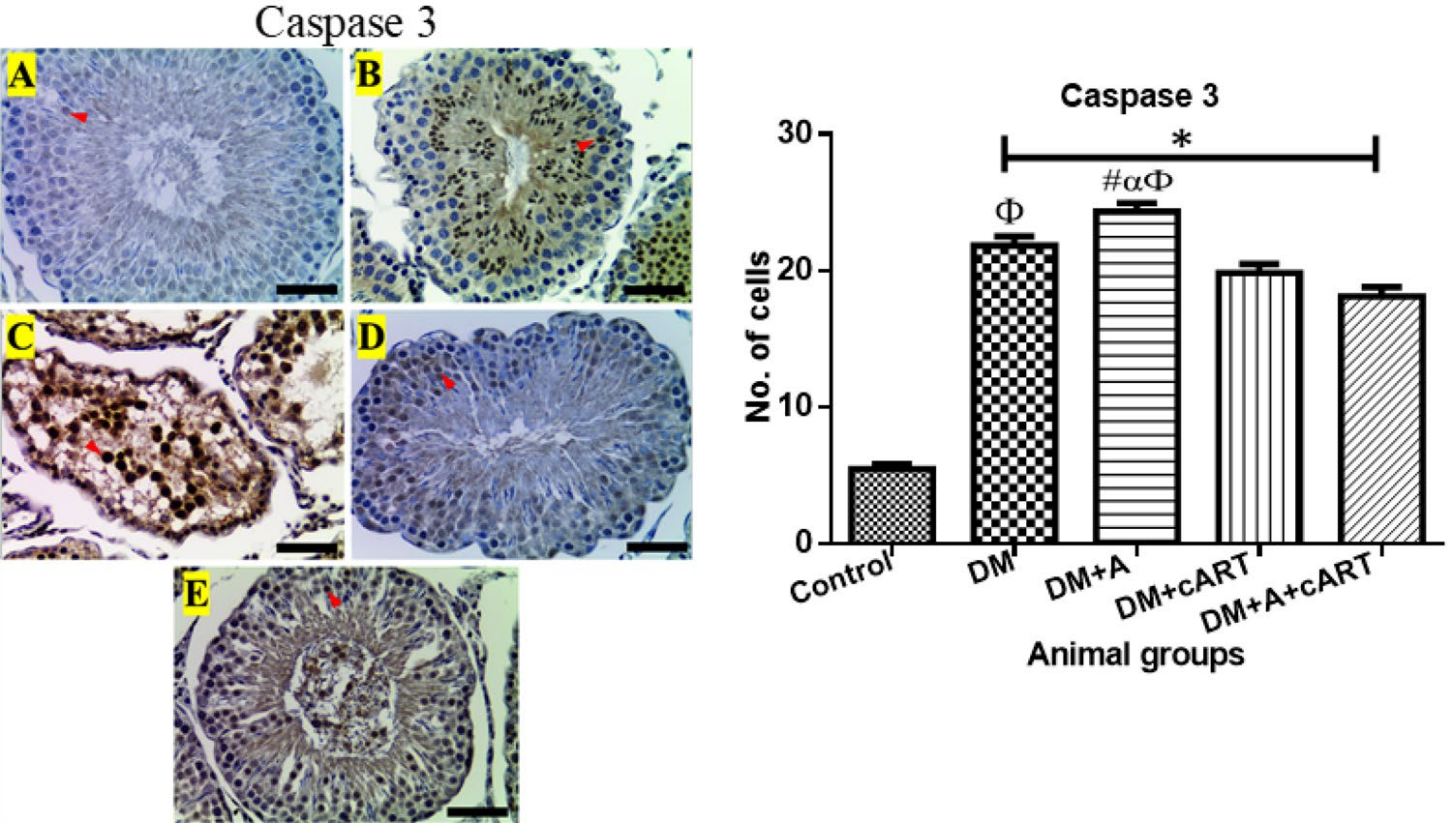
Representative photomicrographs showing caspase 3 expression (red arrowheads) and a graph showing the number of immunostained cells. Different symbols *, #, α, and Ф represent significant differences (*p* < 0.05) as analyzed by a Bonferroni’s multiple comparison test; ‘*’ significantly increased compared to control, ‘#’ significantly increased compared to DM, ‘α’ significantly increased compared to DM + cART, and ‘Ф’ significantly increased compared to DM + A + cART. Magnification, × 400; scale bar, 50 μm. Key: Images in panel: **a** control group, **b** DM group, **c** DM + A group, **d** DM + cART group, and **e** DM + A + cART group. Treatment groups: DM (diabetes); DM + A (diabetes and alcohol); DM + cART (diabetes and combination antiretroviral therapy); DM + A + cART (diabetes and alcohol and combination antiretroviral therapy)

Caspase 3 immunostaining was detected in the germinal epithelium cells of both control and treated groups (Fig. 5). Compared to the control, all treated groups showed a significant increase (*p* < 0.0001) in the number of germ cells expressing caspase 3. However, the expression of caspase 3 was significantly increased in DM + A treated group in comparison with other treated groups (DM: *p* = 0.0385, DM + cART: *p* < 0.0001, and DM + A + cART: *p* < 0.0001). and the expression in DM treated group increased significantly (*p* = 0.0002) compared to DM + A + cART treated group.

### Proliferation marker, Ki-67 immunoexpression

**Fig. 6:**
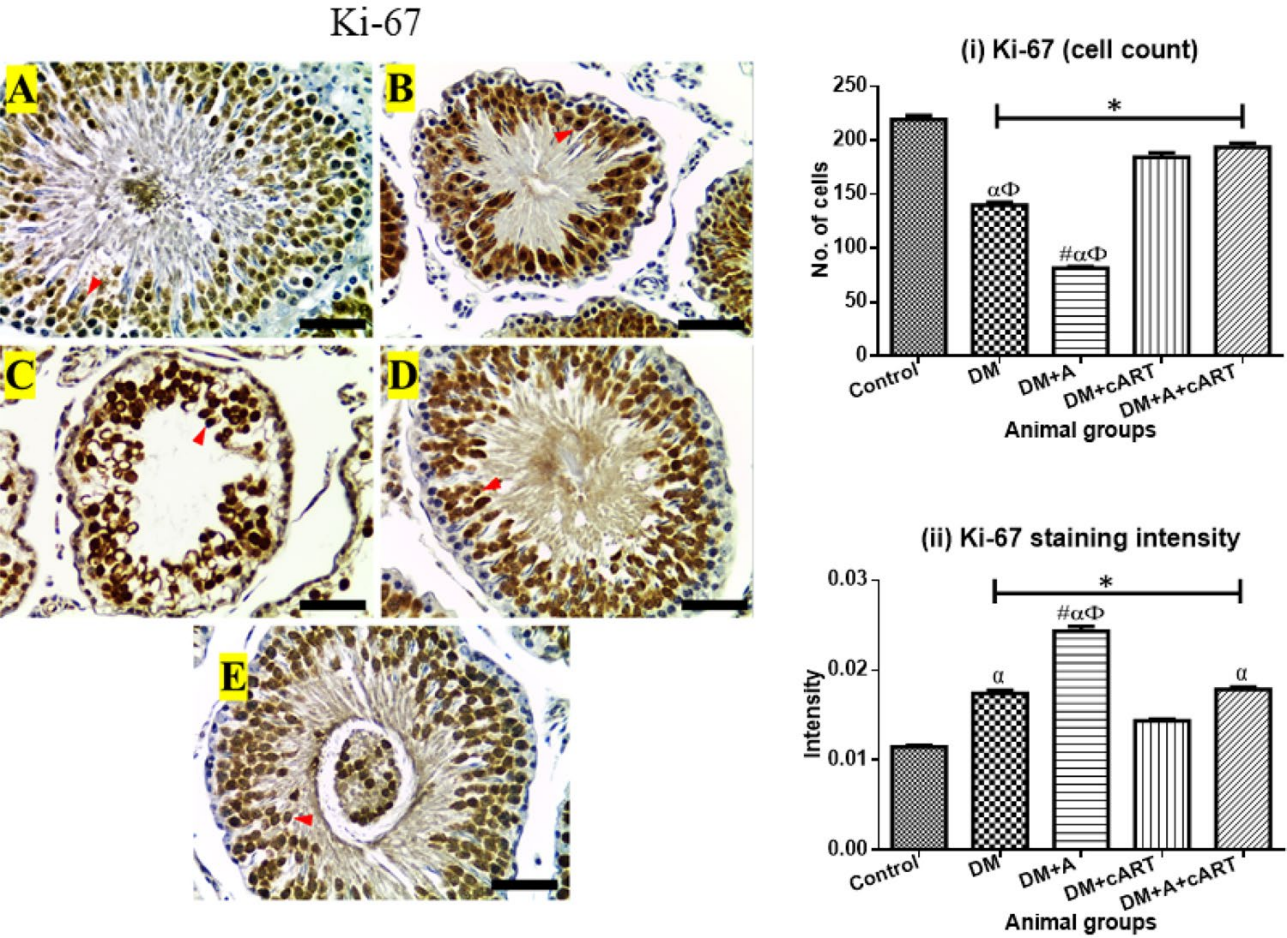
Representative photomicrographs showing Ki-67 expression (red arrowheads) and respective graphs of the number of immunostained cells and staining intensity. Different symbols *, #, α, and Ф represent significant differences (*p* < 0.05) as analyzed by a Bonferroni’s multiple comparison test; ‘*’ significantly different compared to control, ‘#’ significantly different compared to DM, ‘α’ significantly different compared to DM + cART, and ‘Ф’ significantly different compared to DM + A + cART. Magnification, × 400; scale bar, 50 μm. Key: Images in panel: **a** control group, **b** DM group, **c** DM + A group, **d** DM + cART group, and **e** DM + A + cART group. Treatment groups: DM (diabetes); DM + A (diabetes and alcohol); DM + cART (diabetes and combination antiretroviral therapy); DM + A + cART (diabetes and alcohol and combination antiretroviral therapy)

The Ki-67 expression was found in germ cells, majorly the spermatocytes and round spermatids of the control and treated groups (Fig. 6). A significant reduction in the number of germ cells expressing Ki-67 was detected in all treated groups (*p* < 0.0001) in comparison with control. Ki-67 expression in DM + A treated group reduced significantly (*p* < 0.0001) compared to the other treated groups, and the expression in DM group was also significantly reduced (*p* < 0.0001) respectively when compared with DM + cART and DM + A + cART treated groups. However, Ki-67 staining intensity increased significantly (*p* < 0.0001) in all treated groups compared to the control. The intensity of Ki-67 in DM + A group was significantly increased (*p* < 0.0001) in comparison with the other treated groups, and the intensity in DM and DM + A + cART treated groups increased significantly (*p* < 0.0001) compared with DM + cART treated group.

## Discussion

Diabetes and combination antiretroviral therapy (cART) regimen due to HIV infection are a huge public health concern [39], and alcohol abuse as a lifestyle is also prevalent in society [40]. The prevalence of these trio (diabetes, alcohol abuse, and cART use) and their co-existence in one individual is rising in sub-Saharan Africa, especially in males of reproductive age [1–3]. Consistent with previous studies [35, 41], our results showed a decreased food intake but increased fluid intake in treated groups when compared to the control group. Further, the final body weight of all treated groups decreased, resulting in increased gonadosomatic index (GSI), except the DM + A (diabetic and alcohol) treated group which showed a slight GSI decrease compared to control. Both increased and decreased GSI implicate impairment in testicular structure and function [42, 43]. Conversely, increased GSI suggests an increase in testicular weight due to fibrosis and/or inflammation [43], but a decreased GSI reflects a reduction in testis weight that could have resulted from tissue degeneration [42, 44].

In this study, the oxidative stress biomarkers evaluated (inducible nitric oxide synthase (iNOS), malondialdehyde (MDA), and 8-hydroxydeoxyguanosine (8-OHDG)) were significantly upregulated in the testis of all treated groups relative to control. Oxidative stress plays a major role in the pathogenesis of testicular dysfunctions [14, 45], and incidentally, diabetes, alcohol, and cART are well-established oxidative stress inducers [5, 18, 19]. Corroborating with the results of this study, increased expression of iNOS has previously been reported in testis of diabetic rats [46], rats exposed to alcohol [23], and those treated with cART [47]. Additionally, a clinical study by Coştur et al. [48] observed an intense iNOS expression in testis of azoospermic patients. Remarkably, iNOS was greatly upregulated in diabetic animals treated with both alcohol and cART compared to other treated and control groups, suggesting a heightened oxidative stress induction due to alcohol-cART-diabetes interaction.

Earlier studies have established that iNOS plays a key role in induction of testicular oxidative stress through catalysis of nitric oxide production [27, 28, 49]. Though nitric oxide (NO) level was not determined in the present study, the upregulation of iNOS would imply an increase in nitric oxide level [44, 48]. At low concentrations (< 1 µM), NO promotes homeostasis, cell proliferation, and survival, but elevated NO levels (> 1 µM) which may occur following induction of oxidative stress by chemical insults stimulate spermatogenic cell proliferation arrest and apoptosis [24, 49, 50]. This conforms with the significant germ cell loss and distortions of seminiferous tubules observed in the testis of treated animals.

Furthermore, testis tissue has relatively high amounts of unsaturated fatty acids compared to other tissues [29, 30]. In addition, Leydig cell utilizes high amounts of fatty acids during the biosynthesis of testosterone [51] and thus, are very susceptible to lipid peroxidation [52]. Moreover, macrophages which are a main source of iNOS/NO lie adjacent to Leydig cells in the testicular interstitium, making the Leydig cells immediate targets of iNOS/NO activity [28, 48]. Consequently, high testicular iNOS/NO levels stimulate lipid peroxidation leading to production of unsaturated reactive aldehyde, malondialdehyde (MDA) [50, 53]. Accordingly, elevated MDA levels were recorded in all treated groups, the diabetic animals treated with alcohol (DM + A group) had the highest MDA levels. Our findings agree with previous studies that recorded increased MDA levels in testis tissue homogenate of rats that were diabetic [54], exposed to alcohol [6], and treated with cART [32]. However, in the present study, elevated MDA levels were recorded not only in diabetic animals but in all the treatment combinations. Elevation of MDA levels is an evidence of lipid peroxidation and causes testicular cell disintegration, subsequently resulting in impairments of steroidogenesis and spermatogenesis [26, 53, 55].

Additionally, testicular iNOS/NO upregulation stimulates an increased formation of reactive oxygen species (ROS) and lowers cellular antioxidant production [26]. ROS is a potent mediator of DNA oxidation, consequently leading to DNA breakdown and generation of 8-hydroxydeoxyguanosine (8-OHDG) [56]. Conversely, increased nuclear 8-OHDG is an indicator of testicular oxidative stress [14, 45], and has been suggested to induce several mutations such as transitions, deletions, frameshifts, and epigenetic changes that subsequently lead to infertility and genetic disorders in offspring [45, 56]. Therefore, the increased levels of 8-OHDG observed in the testis of treated animal groups suggests severe oxidative stress induced by the treatments. Further, previous studies have reported increased testicular DNA fragmentation in rats treated with alcohol [23] and cART [47], which adversely affect the male reproductive capacity.

Furthermore, earlier studies reported that elevated iNOS/NO levels stimulate testicular inflammation via the nuclear factor- kβ (NF-kβ) pathway [26, 55], leading to the release of cytokines such as interleukin-1β (IL-1β), interleukin-6 (IL-6), tumor necrosis factor-α (TNF-α), and interferons (IFNs) [44, 49]. Although NF-kβ antibody immunostaining was nonspecific (not reported) in the present study, previous studies have demonstrated increased testicular NF- kβ in conditions associated with testicular oxidative stress and inflammation [14, 18]. We found significantly elevated levels of testicular pro-inflammatory cytokines such as IL-1β, IL-6, and TNF-α in the treated groups, except in the DM + A group (diabetic animals treated with alcohol) that showed a significantly decreased IL-6 expression relative to the control group. This suggests induction of testicular inflammation in line with previous reports of elevated levels of proinflammatory cytokines (IL-1β and TNF-α) in testicular tissue homogenate of diabetic rats [54] and those treated with cART [47].

Earlier studies have reported increased levels of pro-inflammatory cytokines in testicular injury, infection, ischemia, and toxicosis [57–59]. Upregulation of testicular cytokines is associated with suppressed steroidogenesis, disruption of blood-testis barrier (BTB) integrity, and diminished spermatozoa viability, which subsequently lead to spermatogenesis and fertility impairments [60, 61]. Furthermore, studies show that both increase and decrease in the expression of IL-1β [62] and TNF-α [63] can be detrimental to Leydig cell function, through inhibition of Leydig cell cytochrome P450 steroidogenic enzymes (CYP11A1 and CYP17A1) [62, 63]. The alternations in testicular cytokines recorded in this study corroborates with earlier reports and conform with the testicular structure and cellular derangements, that will eventually cause steroidogenesis and spermatogenesis failure.

Consequently, accumulation of ROS (oxidative stress) and cytokines (inflammation) are both triggers of testicular cell apoptosis [44, 64]. Our results revealed that immuno-expression of caspase 3, an executioner of cell apoptosis increased significantly in testis of treated groups relative to the control, which implies increased apoptosis due to the treatments. Additionally, previous studies have reported apoptosis in the testis of animals that are diabetic [44, 65], exposed to alcohol [66, 67], and treated with cART [47, 68]. Further, a significant decrease in the number of germ cells expressing Ki-67 but with strong staining intensity was recorded in the treated groups suggesting a disruption of germ cell proliferation and spermatogenesis dysfunction. Similar findings have been demonstrated in cryptorchidism [69] and fluoride-induced testicular toxicity [70].

In conclusion, this study demonstrated that diabetes, alcohol, cART, and their concurrency trigger testicular oxidative stress, inflammation, apoptosis, and disruption of spermatogenic cell proliferation, leading to testis structural and spermatogenesis derangement. Our results suggest that the deleterious impacts of alcohol consumption or/and cART use in diabetic condition on the testis may be mediated through iNOS activity upregulation. The current study highlights the possible critical male reproductive health impairments that may arise amongst diabetic patients who are on cART therapy and consume alcohol regularly.

## Data Availability

The minimal dataset for the results from this study will be made available through a University of the Witwatersrand archived link.
